# Annotations

**Published:** 1902-01-25

**Authors:** 


### ANNOTATIONS.

### Special Modes of Infection.

THE last meeting of the Society of Medical Officers of Health was occupied in considering a paper by Dr. Davies, of Bristol, on the control and exclusion of plague, and it was not without interest to observe how largely the discussion centred itself upon the problem of the rats. Nothing can illustrate more markedly the rapidity with which knowledge grows, or at any rate with which opinions change, than the sudden and almost universal acceptance given to the rat theory of plague. That rats die in large numbers when plague prevails has long been known, nor did it seem altogether strange that this should happen, in view of the peculiar habits of the rat. With man would suffer those animals which live along with man, and among others rats. This seemed reasonable but it led to nothing. But that plague should spread the other way, not from man to rats but from rats to man, and that the rat should be the central factor in the etiology of the disease was another affair, and it is interesting to note, as showing how much more fertile are ideas than facts, that it has only been since the suggestion was made as to the manner in which this rat disease is communicated to man, namely, by fleas and other biting insects, an idea which is by no means proven, but one which makes the facts seem "reasonable" and comprehensible, that the rat theory has come into vogue. Without that idea the mere fact of the association of rats with plague was a sterile bit of knowledge which was practically ignored; with it all seems plain and the whole world agrees that rats are the enemy. What we have now to face is the fact pointed out by Dr. Davies that while the regulations of the Local Government Board, issued so lately as the end of 1896, were well enough as regards the importation of the disease by infected persons, they wholly ignored its introduction by means of rats, especially in ships with cargoes of grain or cotton-seed. Yet this is the real centre of the question. Thousands of infected rats are at present carried from port to port, and freely allowed to infect those on shore. Meanwhile, we carefully isolate every case that occurs among human beings, a practice "as irrational as would be the isolation of every case of hydrophobia in man, while troops of rabid dogs were allowed to remain at large." The old idea that general sanitary measures will protect us from all kinds of diseases is quite exploded. Each malady must be attacked by methods peculiar to itself.

### Faith Healing and Small-pox.

IT is to be hoped that the abominations which were laid bare in regard to the Horos case will have opened the eyes of the British public to the quackeries and iniquities which underlie the various new and fantastic forms of so-called religion which are introduced to notice from time to time. One must not forget, however, while the wickedness lies mostly at the door of the teachers, much harm is done also by the followers—or should we not rather say the dupes—of those creeds which combine with religion the pretence of healing. The old commercial principle of *caveat emptor* is no doubt applicable enough to many new-fangled faiths, and if a man comes across a bad egg in his search for a "religion" congenial to his taste, he has only himself to thank. But when the creed which he picks up teaches him to defy the sanitary laws which have been made for the good of his fellow-citizens, then these "religions" with which we are plagued become a public offence, and require to be suppressed with a firm hand. According to the New York *Medical News* a family in Jersey City was lately discovered with eight of its members suffering from small-pox. The father, although himself suffering from the disease, was treating his children by prayer alone. The eldest daughter died before she could be removed to hospital, and the others were found to be in a serious condition; yet, in spite of their helplessness, and with entire indifference to the danger to which their neighbours were exposed, this infected family resisted the intervention of the health officers. This furnishes us with a good example of the manner in which faith-curing religions do harm to the public, and we see in it a special reason why they should be regarded as anti-social institutions. The capacity for religious belief is an attribute which the highest share with almost the lowest of mankind. When well directed it furnishes the noblest impulse towards good works; when twisted by half criminal and half crazy teachers, Christian scientists, faith healers, and others, it becomes an unmitigated nuisance.

### Official Lymph.

THE Local Government Board have issued a notice stating that although their statutory duty in regard to the supply of glycerinated calf lymph is limited to supplying it to public vaccinators and for purposes of primary vaccination only they so far have not limited themselves to this duty but have furnished lymph to public vaccinators for re-vaccination also. They point out, however, that the supply which they can produce is subject to limitations and that they may find themselves unable to meet all the applications made to them for this purpose. They therefore would be prepared to sanction any necessary expenditure incurred by the guardians of any Poor Law union who, for purposes of re-vaccination may have to supplement from other sources the supply of lymph obtained from the Local Government Board. As an emergency order this is all very proper, but it does not go far enough to render it effectual in cases where the guardians are obstructive. It is to be noted that the circular gives no authority to the public vaccinator to provide himself with lymph at the public expense. It is the Guardians who are to decide whether they will incur the expenditure, and under the present ridiculous arrangement, according to which the protection of the people against small-pox is taken out of the hands of the sanitary authority and handed over to the guardians, these guardians may at their own sweet will leave an open door to small-pox wherever they like. In London even the penalty of having to pay for the small-pox when it occurs is removed from them, for the maintenance of the victims is thrown on the common Poor Fund drawn from the whole metropolis.

